# Variation in the Humoral Immune Response Induced by the Administration of the BNT162b2 Pfizer/BioNTech Vaccine: A Systematic Review

**DOI:** 10.3390/vaccines10060909

**Published:** 2022-06-07

**Authors:** Karen Cortés-Sarabia, Mayralina Gutiérrez-Torres, Escarlet Maleny Mendoza-Renteria, Marco Antonio Leyva-Vázquez, Amalia Vences-Velázquez, Daniel Hernández-Sotelo, Fredy Omar Beltrán-Anaya, Oscar Del Moral-Hernández, Berenice Illades-Aguiar

**Affiliations:** 1 Laboratorio de Inmunobiología y Diagnóstico Molecular, Facultad de Ciencias Químico Biológicas, Universidad Autónoma de Guerrero, Chilpancingo de los Bravo 39086, Mexico; kcortes_sarabia@hotmail.com (K.C.-S.); mayralinagutierrez@hotmail.com (M.G.-T.); escarlet.maleny@gmail.com (E.M.M.-R.); ameliavences.v@uagro.mx (A.V.-V.); 2 Laboratorio de Biomedicina Molecular, Facultad de Ciencias Químico Biológicas, Universidad Autónoma de Guerrero, Chilpancingo de los Bravo 39086, Mexico; leyvamarco13@gmail.com; 3 Laboratorio de Epigénetica del Cáncer, Facultad de Ciencias Químico Biológicas, Universidad Autónoma de Guerrero, Chilpancingo de los Bravo 39086, Mexico; danhs1mx@yahoo.com; 4 Laboratorio de Virología, Facultad de Ciencias Químico Biológicas, Universidad Autónoma de Guerrero, Chilpancingo de los Bravo 39086, Mexico; frebeltran@hotmail.com

**Keywords:** BNT162b2, vaccine, SARS-CoV-2, COVID-19, humoral response, comorbidities, immunosuppression

## Abstract

The BNT162b2 Pfizer/BioNTech vaccine was the first emergency approved vaccine during the COVID-19 pandemic. The aim of this systematic review was to examine the variations in the humoral immune response induced by the administration of the BNT162b2 vaccine in patients with previous SARS-CoV-2 infection, the elderly, and those with comorbidities and immunosuppression states. Additionally, we analyzed the effect of generated neutralizing antibodies against the new variants of concern of SARS-CoV-2. Pubmed, Science Direct, Mendeley, and WorldWide Science were searched between 1 January 2020 and October 2021 using the keywords “BNT162b2”, “serology”, “comorbidity”, “immunosuppression”, and “variants of concern”dA total of 20 peer-reviewed publications were selected. The analysis showed that those individuals with previous infections have a considerably higher antibody response after the administration of BNT162b2 vaccine in contrast with seronegative individuals. With regard to variation in immune responses, elderly individuals, patients with cancer, or patients who had undergone a kidney transplant, dialysis, or who were pregnant had a lower antibody response in comparison to healthy individuals. Finally, antibodies developed against the S protein produced by the BNT162b2 vaccine, possessed lower neutralizing activity against the alpha, beta, gamma, and delta variants of SARS-CoV-2. In conclusion, patients with immunodeficiencies and comorbidities have a lesser antibody response, about which further studies need to be performed in order to analyze the effectiveness and duration of the humoral immunity associated with vaccination in these specific populations.

## 1. Introduction

The COVID-19 pandemic has caused more than 6 million deaths and around 456 million confirmed cases worldwide (https://covid19.who.int/: accessed on 20 May 2022). It is associated with the infection of the newly described SARS-CoV-2 (severe acute respiratory syndrome coronavirus 2), which is transmitted by the inhalation of respiratory droplets during coughing or sneezing, close contact with infected individuals, and aerosol sprays with high viral load [1]. The main symptoms of COVID-19 are fever, cough, fatigue, and difficulty in breathing. Asymptomatic patients have a positive molecular test, but do not present any symptoms that are usually present in patients with mild disease. Moderate disease includes pneumonia-like symptoms such as dyspnea and hyperventilation, whereas severe cases are associated with pneumonia and a respiratory rate higher than 30 breaths/min, severe respiratory distress, and oxygen saturation less than 93%, which could lead to several complications, such as acute respiratory distress syndrome, severe pneumonia, or organ dysfunction commonly associated with death [2]. The multiple clinical manifestations associated with COVID-19 are a consequence of the overactivation of immune response mechanisms that provoke minor or major damage [3].

SARS-CoV-2 is an enveloped single-stranded RNA virus of around 30 kb, structurally it has a diameter of 80-160 nm, and it encodes four major structural proteins, including spike (S), membrane (M), envelope (E), and nucleocapsid (N) [4]. The virus enters into the host cell through the interaction of the S protein, specifically of the receptor binding domain (RBD) in the S1 subunit with the angiotensin converting enzyme 2 (ACE2) [5]. In the intracellular environment, the virus is recognized by the Toll-like receptors 3 and 7 and natural cytotoxicity receptors (NCR), such as NKp46, NKp30, NKp44, NKG2D, DNAM-1, and NKG2C [6], associated with the release of proinflammatory cytokines such as tumor necrosis factor-alpha (TNF-α), interferon-gamma (IFN-γ), interleukin-1 (IL-1) and IL-6, chemokines such as CCL2, CCL8 CXCL2, CXCL8, CXCL9, CXCL16 [7], and Il-8, and regulatory cytokines such as transforming growth factor-beta (TGF-β) [8]. The release of cytokines allows for the recruitment of antigen presenting cells (APC), natural killer (NK) cells, and macrophages to the site of infection [9]. The APC recognize and capture virus-derived antigens for the activation, proliferation, and differentiation of T and B cells. T CD4+ cells release cytokines for the chemiotaxis of T CD8+ cells, which will recognize infected cells to induce apoptosis through the release of perforins and granzymes or by means of the interaction of Fas/FasL, whereas B cells are associated with the specific production of IgM, IgA, and IgG [10]. IgM and IgA appear during the first 5 days after the onset of symptoms [11], whereas IgG can be detected after 12–14 days [12] and continue to circulate until 8 months after infection [13].

The main objective of vaccination is the induction of the humoral immune response to reduce the transmission of diseases [14]. There are four main types: inactivated, attenuated, recombinant proteins, and RNA/DNA using viral vectors [15]. During COVID-19, different types of vaccines have been developed, in which the main target is the S protein of SARS-CoV-2 due to its key role during the infection and the associated immunogenicity [16]. Among these, those vaccines approved for their emergency use by the World Health Organization (WHO) comprise mRNA-1273 (Moderna, Cambridge, MA, USA), BNT162b2 (Pfizer/BioNTech, Mainz, Germany), Ad26.COV2. S (Johnson & Johnson, New Brunswick, NJ, USA), AZD122 (AstraZeneca, Cambridge, UK), Covishield (Serum Institute in the India and AstraZeneca, Cambridge, UK), BBIBP-CorV (Sinopharm, Beijing, China), and CoronaVac (Sinovac, Beijing, China) [14]. The Pfizer/BioNTech BNT162b2 vaccine showed 95% efficacy against COVID-19 [17] and has been approved for its administration in health workers, in persons > 11 years of age, and patients with hypertension, diabetes, asthma, lung, liver, and kidney disease, as well as chronic infections by the human immunodeficiency virus (HIV), the hepatitis C virus (HCV), and the hepatitis B virus (HBV) [18]. The vaccine is administered in two doses of 30 µg each and is composed of a single-stranded mRNA generated by in vitro transcription using as template the DNA that encodes for the S protein of SARS-CoV-2. In addition, to promote delivery into the host cell, the mRNA is encapsulated into lipid nanoparticles. After their administration, transfected cells will produce the S protein, which will be processed and coupled with molecules of the major histocompatibility complex (MHC) for the activation of T CD4+ cells. The latter will promote the activation of B cells for the production of neutralizing antibodies and the activation of T CD8+ cells within the following 7 to 10 days (maxima peak at day 21) after vaccine administration [19]. 

Antibodies generated after the administration of the BNT162b2 vaccine can be detected during the first 12 days [20]. After the boost, an increase in IgG and IgA and a decrease in the titers of IgM antibodies is usually observed [21]. The effectiveness of this vaccine in all types of populations continues to be evaluated, and certain doubts about its efficacy against the new variants of concern and the influence of host-derived factors such as age, comorbidities, and previous infections by SARS-CoV-2, are being studied. Thus, the main objective of this systematic review was to analyze the current information about variations in the humoral immune response induced by the application of the BNT162b2 vaccine in the previously mentioned populations. 

## 2. Materials and Methods

We prepared this systematic review according to the preferred reporting items for systematic reviews and meta-analyses (PRISMA) guidelines and statement recommendations [22]. In this section, we describe all of the information sources and the search strategies employed for obtaining the selected information.

### 2.1. Information Sources and Search Strategy

Relevant publications were identified through electronic searches in ScienceDirect, Mendeley, Pubmed, and WorldWide Science. The search was performed from 17 September to 5 October of 2021, employing the keywords “BNT162b2”, “serology”, “comorbidity”, “immunosuppression”, and “variants of concern” in combination or with the Boolean operator “AND”. The screened period was from 1 January 2020 to October 2021. 

### 2.2. Study Selection

We first screened the titles and abstracts of all preselected articles to remove all duplicates. The remaining articles were independently reviewed by all of the authors and all those files that did not meet our inclusion criteria were removed.

### 2.3. Eligibility Criteria

Published articles that contained the aforementioned keywords or were observed to be related with the main subject were included. We primarily selected articles focused on the evaluation of immune responses in several populations in which the BNT162b2 Pfizer/BioNTech was applied, and also, articles about the evaluation of immune response in individuals with previous infections, comorbidities (cancer), immunosuppression states (pregnant, long-term treatments, and organ transplant), the elderly, and infections by variants of concern of SARS-CoV-2 were selected. Preprints or unpublished as well as articles in any language other than English were excluded.

## 3. Results

### 3.1. Characteristics of the Papers Included

**Figure 1:**
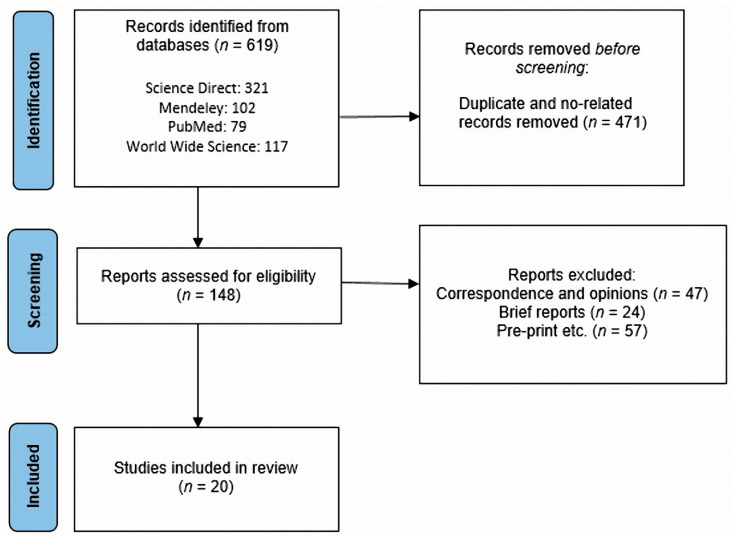
Study identification, PRISMA flowchart.

A total of 20 articles were selected according to the inclusion criteria shown in Figure 1. Articles were based on the immune response evaluation after the administration of the BNT163b2 Pfizer/BioNTech vaccine in several populations. We analyzed the antibody’s appearance time and its possible association with age, sex, comorbidities such as cancer, and immunocompromised states such as pregnancy, long-term treatments, and organ transplantation. We also included articles concerning the neutralizing activity of antibodies against the variants of concern of SARS-CoV-2 (Figure 1).

### 3.2. Mechanism of Action of the BNT162b2 Pfizer/BioNTech Vaccine

**Figure 2:**
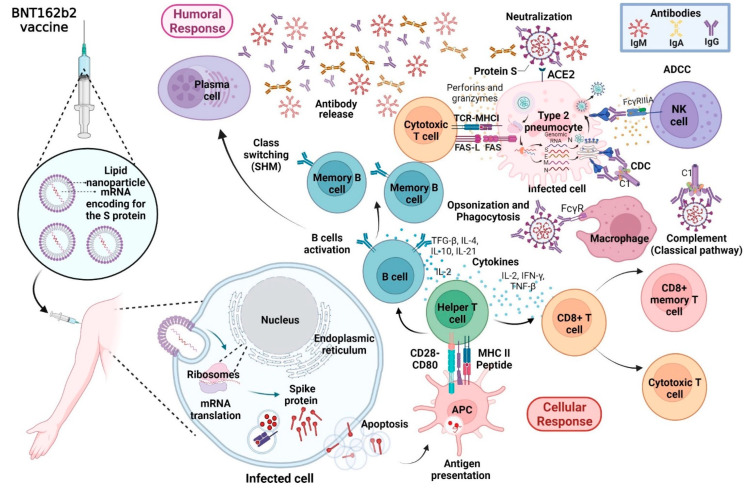
Immune response mechanism after the administration of BNT162b2 Pfizer/BioNTech vaccine. After its administration, the vaccine, which is composed of lipid nanoparticles with encapsulated mRNA, induced the production of the spike glycoprotein of SARS-CoV-2 in the host cells. After its recognition by the immune system, it generates a specific cellular and humoral immune response. Released antibodies promote ADCC, CDC, opsonization, neutralization, and complement activation (classical pathway). ACE2: angiotensin-converting enzyme 2; APC: antigen presenting cell; MHC-I: major histocompatibility complex type I; MHC-II: major histocompatibility complex type II; Tfh: T follicular cell; HSM: hypersomatic mutation; Th1: T helper 1; ADCC: antibody dependent cellular cytotoxicity and CDC: complement dependent dytotoxicity. Image created with Biorender (https://biorender.com/; accessed on 25 May 2022).

The potential immune response mechanism associated with the administration of the BNT162b2 vaccine is briefly illustrated in Figure 2. The serology has been used for the evaluation of COVID-19 vaccines, considering the recombinant RBD derived from SARS-CoV-2 as the main antigen. Patients who received the BNT162b2 vaccine reported the presence of IgM, IgA, and IgG antibodies 12 days after the first shot [20] and, after the second dose, a specific increase in IgA and IgG was observed due to class switch [21]. Despite all of the results derived from the clinical data and the first analysis performed in vaccinated populations, the effectivity of the BNT162b2 vaccine could be affected by multiple factors, among these a clinical history of previous infection or infection with the new variants of concern, age, gender, and the diagnosis of comorbidities and immunosuppression, which are described below.

### 3.3. Factors Associated with the Variation of Immune Response

#### 3.3.1. BNT162b2 Vaccine and Previous Infections

During the initial evaluations of the humoral immune response induced by the application of the BNT162b2 vaccine in countries such as the United States, Italy, Belgium, Israel, the United Kingdom, Canada, and Japan, the authors provided information regarding the effect of previous infections on the antibody positivity rate after the application of one or two doses of the vaccine. Fraley et al. [23] in the U.S.A, using a binding assay for the detection of IgG and IgA against the proteins S and N of SARS-CoV-2, after the application of two doses in a population of 193 individuals, among whom 42 participants had previous infections by SARS-CoV-2, reported a higher positivity rate after the first dose in those individuals with previous infections. Similar results were observed by Favresse et al. [24] in Belgium, on evaluating 200 individuals, among whom 58 reported previous infections. In healthcare workers from Italy, Padoan et al. [25], using 163 samples, of which 15 were previously infected, revealed that seropositive individuals have significant IgG values in comparison to infection-naïve subjects 12 days after vaccine application, whereas Efrati et al. [26] in Israel and Tauzin et al. [27] in Canada demonstrated that a single dose of an mRNA-based vaccine could be sufficient to induce antibody response and that neutralizing antibodies are higher in seropositive individuals. In conclusion, all of the aforementioned articles reported an increase in the immune response after the application of one or two doses of the BNT162b2 vaccine in individuals who reported previous infections or who tested seropositive in comparison with individuals that were seronegative (Table 1).

#### 3.3.2. BNT162b2 Vaccine and Age/Gender

As people grow older, several systems are affected, including the immunological system, and that could be associated with the effectiveness in the immune response induction after vaccination. Bayart et al. [28], in their study performed in individuals aged >40 years who received the BNT162b2 vaccine, observed an inverse association between the antibody titers and age (increased age, fewer antibodies titers); also, the authors reported the presence of lower antibody titers in men after 42 days. Similar results were obtained by Michos et al. [29], who reported the presence and a higher neutralizing activity in young people without a significant difference according to gender, whereas Lo Sasso et al. [30] and Jalkanen et al. [31] described lower antibody titers in older individuals (aged > 50 years), with a higher neutralizing activity in women than in men (Table 2).

#### 3.3.3. BNT162b2 Vaccine and Comorbidities

Initial studies of COVID-19 reported a greater predisposition to develop severe clinical disease in individuals with comorbidities such as cancer and chronic kidney disease or immunosuppression, as well as during pregnancy, organ transplantation, and long-term treatment [32]. Addeo et al. [33] evaluated the presence of IgG antibodies against the RBD of SARS-CoV-2 after the administration of the BNT162b2 vaccine in patients with cancer (hematological and solid tumors), and the authors observed that antibodies could be detected 3 weeks after application of the second dose. A similar study was performed by Shmueli et al. [34] in Israel; the results of these authors showed a lesser antibody response in individuals with cancer in comparison with immunocompetent individuals after administration of the dose. In relation to immunosuppression states, such as pregnancy, Bookstein et al. [35] reported that pregnant women have a lower antibody response after vaccination in comparison to nonpregnant women. In contrast, Beharier et al. [36] reported that pregnant women with previous SARS-CoV-2 infection developed a faster humoral response after vaccination, whereas in patients with hemodialysis, Grupper et al. [37] detected higher antibody titers in young individuals with shorter treatment time in comparison with older individuals. Finally, Rozen-Zvi et al. [38] studied patients who received kidney transplantation and reported a low antibody response (36.4%) against the S protein in their study group (Table 3).

#### 3.3.4. BNT162b2 Vaccine and Variants of Concern

The World Health Organization (WHO) designated as variants of concerns (VOC) those variants that spread widely and that are more transmissible, causing more severe disease or reducing neutralizing activity. Current VOC comprise: Alpha (B.1.1.7), Beta (B.1.351), Gamma (P.1), Delta (B1.617.2), and the newly described Omicron (B.1.1.529) [39]. To test the effectiveness of the BNT162b2 vaccine against VOC, some studies have been performed. Charmet et al. [40] reported a vaccine effectiveness of 88% to the wild strain, and a similar percentage to the Alpha and Beta variants; however, a reduced effectiveness against the Gamma (77%) VOC was observed. Additionally, López-Bernal et al. [41] reported an effectiveness of 93.7% against the Alpha variant and a diminution to 88% against the Delta variant.

Another interesting characteristic to evaluate is the neutralizing capacity of the antibodies generated by the administration of the BNT162b2 vaccine against the previously reported VOC. Wang et al. [42] in the U.S. reported a reduced neutralizing activity against the Alpha and Beta variants, whereas Tut et al. [43] and Davis et al. [44] in the United Kingdom, and in independent studies, both reported a reducing activity against the Beta, Gamma, and Delta variants of the serum samples derived from patients who received the BNT162b2 vaccine (Table 4).

## 4. Discussion

The BNT162b2 vaccine was the first vaccine approved for its emergency use by the WHO. Despite the favorable results observed in the clinical trials and during the first year after its administration, it is important to continue to evaluate the immunogenicity and associated factors that could be involved in the effectiveness of the vaccine in populations worldwide. Among these, the effect of previous infections, age, gender, comorbidities or immunosuppression states, and the newly described variants of concern of SARS-CoV-2 [21] should be studied. These data could be useful to verify the effectiveness in populations that were not considered in the clinical trial. 

First, antibody responses and IgG levels could be influenced by natural infection, sex, and age [45]. In relation to previous infections with SARS-CoV-2 in patients who received the BNT162b2 vaccine, our selected articles were published during January to October 2021, and most of them were the first reports about this specific subject. However, the authors stated some limitations such as the small number of infected individuals, the analyzed time prior to vaccine application, or the lack of measurement of neutralizing capacity. Those limitations could be due to the conception of the study, the lack of samples or methods to perform complementary analysis, or the haste of publication. To date, newly published articles have provided more evidence about the potential effect of previous infections and BNT162b2 vaccine that complement the aforementioned information as we describe below. 

Initial studies reported that previously infected patients had the highest antibody titers and neutralizing activity against the S protein [46,47] by boosting the quantity, quality, and breadth of the humoral response [48]. Moreover, natural infection was associated with longer and stronger protection against reinfections and hospitalization [49], this hypothesis was later supported by Dhumal et al. [50] and Abu-Raddad et al. [51], in which a low probability of reinfection during the second waves of infection after vaccine application and natural infection was reported. This information could be a guiding factor in the design of more effective vaccination schemes for the future. In relation with the duration of the immune response, short-term immunity (around 6 months) against SARS-CoV-2 infection, it is generated by the application of only two doses of BNT162b2 in naïve-infected individuals; however, immunity in vaccinated individuals with previous infection remained during 10 months to 1 year after post-COVID-19 infection following one or two doses of the BNT162b2 vaccine [52,53]. After the diminution in antibody titers against the virus, components of the adaptive immune response, such as B cells, T CD4+, and T CD8+ cells, can persist during months or even years [54], and can confer protection against reinfections with new variants of SARS-CoV-2.

On the other hand, the immune response has variations associated with age, Gruppel et al. [55] performed the evaluation of IgG antibodies against the S protein in individuals without previous infections after the administration of the BNT162b2 vaccine. All of these developed antibodies 14 days after vaccine application; however, the highest titers were observed in younger individuals in comparison to older individuals (aged > 50 years). Collier et al. [56] reported similar results; however, their studied age group was around 72 years. The authors concluded that age and antibody response have an inverse relation; remarkably, a significant diminution in antibody titers was observed in older individuals (aged > 80 years). Another interesting fact is the combined effect of previous infections and age on antibody response after the application of the BNT162b2 vaccine. In regard to this subject, an interesting study was performed in the United Kingdom. The authors included elderly patients with and without previous infection; after the administration of the first dose, immune response was evaluated, and it was observed that individuals without previous infections reported a lower adaptive immune response [43]. Similar results were reported in an independent study performed by Wei et al. [57].

Severe COVID-19 is common in individuals with comorbidities such as cancer or chronic kidney disease and with immunosuppression states such as pregnancy, organ transplantation, and drugs and long-term treatments such as hemodialysis [32]. In regard to organ transplantation, Rabinowich et at. [58] studied liver-transplant recipients and reported a lower antibody response (47.5%), which was associated with age and the administered treatment in this group. On the other hand, Schramm et al. [59] reported that cardiothoracic-transplant recipients had neither a detectable humoral nor a cellular response 3 weeks after the administration of the BNT162b2 vaccine. In our study, we did not include studies that evaluated patients with autoimmune diseases; however, due to their imbalance in the immune response, variations in the humoral and humoral response can be detected. Furer et al. [60] evaluated the seropositivity rate in patients with autoimmune inflammatory rheumatic diseases and reported significantly lower IgG antibody levels, which could be due to the administration of glucocorticoids, rituximab, and abatacept, which are commonly used as treatment. Individuals with the aforementioned conditions could have variation in the immune response after the administration of the BNT162b2 vaccine, for which reason it is important to evaluate the fluctuation of the immune response against the S protein after vaccination specifically in this population. 

Finally, the diversification of SARS-CoV-2 has permitted the emergence of new variants with specific mutations associated with increased transmissibility, virulence, and immune response evasion [61]. Abu-Raddad et al. [62] reported an effectiveness of 87% and 72.1% against the Alpha and Beta variants, respectively, whereas Leier et al. [63] reported a 4.3- to 6.5-fold increase in neutralizing activity against Alpha and Beta variants in individuals who recovered from SARS-CoV-2 and received the BNT162b2 vaccine. In relation with the newly described Omicron variant, Collie et al. [64] reported 70% effectiveness; in contrast, Lu et al. [65] searched for the presence of neutralizing antibodies against the HKU691 and HKU344-R346K strains of the Omicron variant in serum samples derived from individuals who were recipients of the BNT162b2 vaccine, and reported an effectiveness of 20% and 24%, respectively. To date, authorized vaccines have been distributed with success to all countries in the world; however, their effectiveness against new variants remains under evaluation [66]. 

## 5. Conclusions

During the past 2 years, the COVID-19 pandemic has been associated with the death of thousands of persons. Approval of vaccines for their emergency use provided a new insight for the reduction in the number of cases or severe disease; among these vaccines, the BNT162b2 vaccine was first administered in health workers and susceptible individuals. Initial clinical studies reported high effectiveness in the production of neutralizing antibodies after administration of the BNT162b2 vaccine; however, real-world effectiveness studies revealed a reduced effectiveness due to some host-derived factors. In this study, we reviewed the effectiveness of the BNT162b2 vaccine in individuals with previous infections, the elderly, and comorbidities and immunosuppression, and reported that individuals with previous infection have a more robust antibody response after vaccine administration, and that factors such as age, gender, cancer, and pregnancy and dialysis are associated with a lower antibody response and neutralizing activity. 

Other interesting groups requiring further analysis are patients with autoimmune diseases (arthritis, type I diabetes, lupus, etc.), and children and adolescents who received the BNT162b2 vaccine in order to test effectiveness and variations in the immune response after the vaccine’s administration. Additionally, new studies need to be designed with the purpose of performing a more robust analysis of the effectiveness of the BNT162b2 vaccine during homologous and heterologous booster vaccination and its effectiveness against the new variants of concern to establish their current use for future booster doses or the need for future changes in design, considering epidemiological reports in the future.

## Figures and Tables

**Table 1:** Antibody response induced by the application of the BNT162b2 vaccine in previously infected individuals.

Author	Country	Participants	Previously Infected Individuals	Doses	Sample	Serological Assay	Results
Fraley et al. [23]	USA	193 (142 women and 51 men)	42	Two	Serum and plasma	Bead-based multiplex assay to IgA and IgG against viral S and N proteins.	↑ Seropositive individuals after the first dose
Favresse et al. [24]	Belgium	200 (55 women and 45 men)	58	Two	Blood	Anti-SARS-CoV-2 nucleocapsid ECLIA and Anti-SARS-CoV-2 spike protein ECLIA.	↑ Seropositive individuals
Padoan et al. [25]	Italy	163 (114 women and 49 men)	13	Two	Serum	Anti-SARS-CoV-2 S-RBD IgG and anti-SARS-CoV-2 S CLIA.	↑ Seropositive individuals 12 days after the first dose
Efrati et al. [26]	Israel	255 (124 women and 131 men)	78	Seropositive individuals had one dose and seronegative had both	Serum	Anti-S1 and Anti-S2 IgG CLIA.	↑ Seropositive individuals after the first dose compared to seronegative after the second dose
Tauzin et al. [27]	Canada	48 (31 women and 17 men)	24	One	Plasma	Anti-SARS-CoV-2 RBD IgM, IgA and IgG ELISA	↑ Neutralizing antibodies in seropositive individuals
						Virus neutralization assay.	

CLIA: chemiluminescent immunoassay; ECLIA: electrochemiluminescent immunoassay; ELISA: enzyme linked immunosorbent assay; ↑: increase.

**Table 2:** Effect of age and gender on the antibody response induced by the BNT162b2 vaccine.

Author	Country	Participants	Age *	Doses	Sample	Serological Assay	Antibody Titers According to Age	Antibody Titers According to Gender
Bayart et al. [28]	Belgium	221 (169 women and 52 men)	43	Two	Blood	CMIA quantitative anti-RBD IgG and ECLIA anti-N.	↓ As age increases.	↓ In men than women 42 days after of the first dose
Michos et al. [29]	Greece	268 (211 women and 57 men)	43	Two	Serum	ECLIA semiquantita-tive anti-S1 and neutralizing ab anti-RBD ELISA kit.	↑ Total and neutralizing antibodies in younger participants.	Without association
Lo Sasso et al. [30]	Italy	2607 (1243 women and 1364 men)	50–60	Two	Serum	CLIA anti- S-RBD IgG.	↓ As age increases.	↑ In women than men
Jalkanen et al. [31]	Finland	230 (182 women and 148 men)	43	Two	Serum	EIA anti-S1 IgG, IgM and IgA.	↓ Anti-S1 IgG antibody levels and neutralization titers in the older age group (55–65 years).	↑ Neutralizing antibodies in vaccinated women

CLIA: chemiluminescent immunoassay; ECLIA: electrochemiluminescent immunoassay; ELISA: enzyme linked immunosorbent assay; CMIA: chemiluminescent microparticle immunoassays; EIA: enzyme immunoassay; ↑: increase; ↓: decline. * average in years.

**Table 3:** Effect of comorbidity and immunosuppression in the antibody response induced by the administration of BNT162b2 vaccine.

Author	Country	Comorbidity/Immunosuppression	Serological Assay	Results
Addeo et al. [33]	USA and Sweden	Cancer (hematologic and solid tumors)	ECLIA anti-RBD IgG against SARS-CoV-2	Antibody detectable levels until the third week after the second dose
Shmueli et al. [34]	Israel	Cancer	ELISA anti-RBD IgG against SARS-CoV-2	↓ Anti-RBD IgG in cancer patients compared with immunocompetent individuals after the first dose
Bookstein et al. [35]		Pregnancy	CLIA anti-RBD IgG against SARS-CoV-2	↓ Antibodies in pregnant compared to nonpregnant women
Beharier et al. [36]		Pregnancy	MBBAs anti-N and anti-S IgM/IgG against SARS-CoV-2	Pregnant women with previous infection develop a faster humoral response
Grupper et al. [37]		Maintenance hemodialysis	CMIA anti-S IgG against SARS-CoV-2	↑ Anti-S IgG in younger patients with less time on dialysis treatment
Rozen-Zvi et al. [38]		Kidney transplant recipients	CMIA anti-S IgG against SARS-CoV-2	↓ Antibody response after vaccination in renal recipients

CLIA: chemiluminescent immunoassay; ECLIA: electrochemiluminescent immunoassay; ELISA: enzyme linked immunosorbent assay; CMIA: chemiluminescent microparticle immunoassays; EIA: enzyme immunoassay; MBBAs: multiplex bead binding assays; ↑: Higher, ↓: Lower.

**Table 4:** Neutralizing capacity of antibodies generated by the administration of BNT162b vaccine against VOC of SARS-CoV-2.

Author	Country	BNT162b2 Vaccine Effectiveness (%)		
		Wild	Alpha	Beta
Charmet et al. [40]	France	88%	86%	86%
Lopez-Bernal et al. [41]	United Kingdom	-	93.70%	-
Wang et al. [42]	USA	-	↓	↓

-: unknown; ↓: reduced neutralizing activity.

## Data Availability

Data are contained within the article.
